# Synthesis of *Cordyceps fumosorosea-*Biochar Nanoparticles and Their Effects on Growth and Survival of *Bemisia tabaci* (Gennadius)

**DOI:** 10.3389/fmicb.2021.630220

**Published:** 2021-02-18

**Authors:** Xingmin Wang, Jing Xu, Tingfei Sun, Shaukat Ali

**Affiliations:** Key Laboratory of Bio-Pesticide Innovation and Application, Engineering Research Centre of Biological Control, College of Plant protection, South China Agricultural University, Guangzhou, China

**Keywords:** *Cordyceps fumosorosea*, microbial pesticides, nano-formulation, *Bemisia tabaci*, toxicity

## Abstract

Nanotechnology can offer an environmentally sustainable alternative to synthetic chemicals for pest management. Nano-formulations of different microbial pest control agents have been effective against several insect pests. Synthesis of *Cordyceps fumosorosea*-biochar (BC) nanoparticles and their bio-efficacy against *Bemisia tabaci* was observed during this study. The characterization of *C. fumosorosea-*BC nanoparticles through different analytical techniques showed successful synthesis of nanoparticles. UV spectroscopy showed a characteristic band of surface plasmon between 350 and 400 nm; SEM images confirmed the synthesis of spherical shaped nanoparticles; X-ray diffractogram showed strong peaks between 2θ values of 20°–25°; and atomic force microscopy (AFM) analysis revealed particle size of 49.151 nm. The bioassay studies demonstrated that different concentrations of *C. fumosorosea-*BC nanoparticles caused significant reduction in hatchability of *B. tabaci* eggs as well as survival of immatures emerging from treated eggs when compared with controls. The results also revealed that *C. fumosorosea-*BC nanoparticles were highly pathogenic against 2nd and 3rd instar nymphs and pupae of *B. tabaci* having LC_50_ values of 6.80, 7.45, and 8.64 ppm, respectively. The LT_50_ values for 20 ppm concentration of *C. fumosorosea-*BC nanoparticles against 2nd and 3rd instar nymphs, and pupae of *B. tabaci* were 3.25 ± 0.29, 3.69 ± 0.52, and 4.07 ± 0.51 days, respectively. These findings suggest that *C. fumosorosea-*BC nanoparticles can potentially be used in biorational *B. tabaci* management programs.

## Introduction

The use of nanotechnology can change the future of both agricultural and food industries by producing novel products/nanoparticles with a wide range of applications (Chinnamuthu and Boopathi, 2009). Nanoparticles may occur in different particulate systems (monomeric, oligomeric, or polymeric) with a specific size and unique physical properties (e.g., uniformity and conductance) so making them worth using in material as well as biological sciences (Moraru et al., 2003). Nanoparticles have been applied within the agricultural sector for different purposes such as to increase soil fertility, control weeds, and protect plants from insects and diseases (Tarafdar et al., 2013). Nanoparticles can be used in different ways and could serve as an efficient tool for insect pest management (Goswami et al., 2010; Bhattacharyya et al., 2016). The nano-encapsulation of pesticides into nano-sized molecules of active pesticide compounds has different advantages like protection of the active ingredients from degradation, improved efficacy and reduced pesticide application to at least 10–15 times less than those applied with classical formulations (OECD and Allianz, 2008).

Insect pathogenic fungi have been considered as potential agents for the biological control of different insects for over a century. *Cordyceps fumosorosea* (previously known as *Isaria fumosorosea*) is a well-known entomopathogen which has been commercialized for the management of different insect pests (Ali et al., 2010a, b). Under favorable conditions, *C. fumosorosea* can cause a significant reduction in insect pest populations (Huang et al., 2010). Apart from its efficacy and low cost of production, the use of *C. fumosorosea* has other advantages including broader insecticidal activity, a diversified host range, and safety for humans and other non−target organisms (Ali et al., 2017). However, *C. fumosorosea* is slow to take effect following practical application and can easily be affected by its environment. Recently, nanoparticles of *C. fumosorosea* have been reported as effective biopesticides against different insect pests (Banu and Balasubramanian, 2014; Wang et al., 2019).

Biochar (BC) produced through biomass pyrolysis under reduced oxygen supply is known to be a rich source of carbon (Hussain et al., 2016). The physical properties of biochar [porous structure, high surface area, and abundant oxygen-containing functional groups such as hydroxyl (–OH) and carboxyl (–COOH) on its surface] as well as low cost of production make it a suitable material for synthesis of nanoparticles (Yao et al., 2013; Zhou et al., 2014).

In the above context, the current investigations were carried out to synthesize and characterize the *C. fumosorosea*-BC nanoparticles in conjunction with studies on the bio-efficacy of nanoparticles against *Bemisia tabaci* Middle East-Asia Minor 1(MEAM 1) cryptic species (formerly ‘B biotype) which is a key pest of different crops and vegetables worldwide along with serving as vector of many plant viruses (Naranjo et al., 2010). The main objectives of the current work were therefore to: (a) synthesize and characterize *C. fumosorosea*-BC nanoparticles through different standardized analytical techniques; and (b) to study the dose mortality responses of *B. tabaci* to *C. fumosorosea*-BC nanoparticles.

## Materials and Methods

### Fungal Inoculum

*Cordyceps fumosorosea* strain SP535 (deposited at Guangdong Microbial Culture Center, Guangzhou, China under repository number GDMCC60514) was cultured on potato dextrose agar (PDA) plates following the method of Ali et al. (2009). The basal fungal suspension (1 × 10^8^ conidia/ml) to be used during the current experiments was prepared using the method of Ali et al. (2017). Briefly, conidia were harvested from PDA plates with deionized water containing 0.03% Tween-80 and sieved using filter paper (Whatman no. 2; Science Kit and Boreal Laboratories, New York, NY, United States) into sterile vials. Conidia were counted using a compound microscope and a hemocytometer (0.0625 m^2^; Fuchs-Rosenthal Merck Euro Lab, Darmstadt, Germany) to calibrate a suspension of 1 × 10^8^ conidia/ml.

### Insect Cultures

*Bemisia tabaci* was reared on *Solanum melongena* as outlined by Wang et al. (2019). The stock of MEAM1 whiteflies were collected in Guangzhou from cotton plants and reared at the Engineering Research Centre of Biological Control, Ministry of Education, South China Agricultural University. The MEAM1 *B. tabaci* were maintained on eggplants in a glasshouse. The plants were cultured in 18.0 cm pots and kept in cages to avoid any premature infestation from whiteflies or other insects until use. The rearing conditions were set at 26 ± 1°C, 70 ± 10% R.H., and a photoperiod of 12 h of light: 12 h of darkness.

### Preparation of *Cordyceps fumosorosea*-BC Nanoparticles

*Cordyceps fumosorosea*-BC nanoparticles were synthesized extra-cellularly following Amerasan et al. (2016) and Wang et al. (2019). Five milliliters of fungal suspension (1 × 10^8^ conidia/ml) was inoculated to freshly sterilized 50 ml PDA broth (in five individual 250 ml Erlenmeyer flasks) and incubated at 150 rpm, 26 ± 2°C for 72 h. Following 72 h, fungal biomass was separated from the broth via filtration across Whatman filter paper #01 and washed three times with ddH_2_O to remove any debris. Ten grams of fungal mycelia was then inoculated into the sterilized ddH_2_O (100 ml) and then incubated again on the rotary shaker at 150 rpm, 26 ± 2°C for 72 h. The fungal broth was again filtered after 72 h and biochar (0.1 g) was added to the culture filtrates and again incubated at 120 rpm, 26 ± 2°C for 72 h. The extra-cellularly prepared culture was stored in a refrigerator until required.

### Characterization of *Cordyceps fumosorosea*-BC Nanoparticles

The extracellular synthesis of *C. fumosorosea*-BC nanoparticles was characterized through using different analytical techniques used for nanoparticle characterization in previous studies (Banu and Balasubramanian, 2014; Amerasan et al., 2016; Wang et al., 2019).

To perform scanning electron microscopy, *C. fumosorosea*-BC nanoparticles were processed and fixed by following the method of Wang et al. (2019). The nanoparticles produced were washed three times with 0.1M potassium phosphate buffer (pH 7.2). The material was then fixed with 2.5% glutaraldehyde in PBS buffer for 3 h at 4°C then rinsed twice with PBS for 10 min each time followed by rinsing with ddH_2_O. The samples were then placed on glass cover slips (5 × 5 mm) and freeze dried at −80°C for 3 h followed by overnight drying at 4°C. The samples were then gold sprayed. The images were captured under SU8010 (Hitachi, Japan) SEM operating at an accelerated voltage of 5.0 kV.

The samples for High Resolution Transmission Electron Microscopy (HRTEM) were prepared by applying a drop of the aqueous suspension of films on carbon coated copper grids under suitable conditions. The sample, grounded into small pieces, was added to liquid nitrogen so that the depth of the resolution could be improved. A JEOL JEM 1200 EXII microscope attached with a UHR pole piece and a lattice resolution of 0.14 nm with the accelerating voltage of 120.0 kV was used to determine the image of the nanomaterials.

The ultraviolet characterization of *C. fumosorosea*-BC nanoparticles was performed by UV-spectroscopy at different wavelengths (300, 400, 500, and 600 nm) in a Nanodrop one Spectrophotometer (Thermo Fisher Scientific, United States). EDX spectroscopy was performed for further characterization of structure as well as composition of *C. fumosorosea*-BC nanoparticles. X-ray diffractometry analysis was undertaken (using Cu-Kα radiations in a Bruker D8 diffractrometer, Germany) for the calculation of the crystalline structure of *C. fumosorosea*-BC nanoparticles. The *C. fumosorosea*-BC nanoparticles were characterized qualitatively through FTIR analysis using a MIR8035 FTIR spectrometer (Thermo Fisher Scientific, Germany).

Atomic force microscopy (AFM) analysis was done to study the surface topology. On a glass slide, 100 μL of the respective sample was placed, in order to get a thin film of the sample. The sample was then allowed to dry for 5 min. AFM Dimension Icon-Bruker, Germany was instrumental in scanning the slides.

Zeta potential (surface charge) was analyzed for both biochar and *C. fumosorosea*-biochar nanoparticles by using the Zetasizer (Malvern, United Kingdom) at 25°C.

### Toxicity of *C*. *fumosorosea*-BC Nanoparticles Against Whitefly

#### Effects of *C*. *fumosorosea*-BC Nanoparticles on Egg Hatchability and Nymphal Development of *B. tabaci*

The detached leaf assay method of Can et al. (2017) was employed to study the influences of *C. fumosorosea*-BC nanoparticles on egg hatchability and nymphal development of *B. tabaci*. Briefly, six weeks old eggplant seedlings (at four to five leaf stage) were used during this study. The homogenous egg production of *B. tabaci* on plant leaves was achieved by releasing five *B. tabaci* adults (three females and two males) via an aspirator into a micro-cage (diameter: 3.5 cm; height: 3 cm) attached to the lower surface of eggplant leaves and a cohort of 50 eggs was marked with indelible ink after 48 h of egg laying. Five concentrations of *C. fumosorosea*-BC nanoparticles (5, 7.5, 10, 20, and 50 ppm) were sprayed to run-off on leaves on whose surface had marked eggs. Whitefly eggs sprayed with ddH_2_O served as untreated control whereas treatment of whitefly eggs with conidial suspension of *C. fumosorosea* (100 ppm) was used as positive control. The complete experimental setup was incubated at 25 ± 1°C; 80 ± 10% relative humidity and 14 h light:10 h dark photoperiod. A few drops of ddH_2_O were added daily to prevent desiccation. The number of hatched eggs were monitored on a daily basis. Percentage egg hatchability was calculated by following Wang et al. (2019) as follows:

    Percent⁢egg⁢hatchability=(Number⁢of⁢eggs⁢hatched/Total⁢number⁢of⁢eggs)×100

The position of newly hatched nymphs was marked with indelible ink following their settling on the leaves. The survival of nymphs was observed on a daily basis which was based on a preliminary test on the developmental period using this host plant (Ali et al., unpublished data). The percentage nymphal mortality was calculated using the following equation:

$$\begin{array}{l} \text{ Percentage mortality}=\\ \text{(Total number of nymphs}-\text{Number of survivingnymphs/}\\ \text{ Total number of nymphs)}\times \text{100} \end{array}$$

#### Microscopic Examination of Egg Infection by *C*. *fumosorosea*-BC Nanoparticles

The mechanism of reduction in the hatchability of *B. tabaci* was studied by observing the *B. tabaci* eggs treated with *C*. *fumosorosea*-BC nanoparticles and control treatments under a Zeiss Stereo Discovery V20 Microscope after 1, 3, 5, and 7 days of infection.

#### Toxicity of *C. fumosorosea*-BC Nanoparticles Against Immature Instars of *B. tabaci*

Six weeks old eggplant seedlings (at four to five leaf stage) were used during this study. The homogenous cohorts of *B. tabaci* immature life stages (2nd and 3rd instar nymphs and pupa) were reared by following Can et al. (2017) as described in the previous section. The whitefly individuals in a cohort (*n* = 30) of five immatures per leaf were demarcated with indelible ink on individual plant leaves for ease of experimentation. Each immature whitefly life stage was sprayed with five concentrations of *C. fumosorosea*-BC nanoparticles (5, 7.5, 10, 20, and 50 ppm) to run-off using an atomizer sprayer for 30 s. Whitefly immatures sprayed with ddH_2_O served as untreated controls whereas treatment of whitefly immatures with *C. fumosorosea* conidial suspension (100 ppm) were used as positive controls. The complete experimental setup was incubated at 25 ± 1°C; 80 ± 10% relative humidity and 14 h light:10 h dark photoperiod. The whole experiment was repeated six times. The mortality of immatures was recorded on a daily basis until 5 days post treatment when median lethal concentration (LC_50_) was calculated through probit analysis (Finney et al., 1971). Furthermore, *C. fumosorosea*-BC nanoparticles were also tested against different immature life stages of whitefly (*n* = 30) at 20 ppm on eggplant leaves as described above to calculate the median lethal time (LT_50_) after 7 days of treatment. The immature life stages treated with *C*. *fumosorosea*-BC nanoparticles were observed under a Zeiss Stereo Discovery V20 Microscope after 1, 3, 5, and 7 days of infection.

### Statistical Analysis

Data regarding egg hatchability and survival of immatures were analyzed through ANOVA-1 and significant differences between means were calculated through Tukey’s HSD test (*P* ≤ 0.05). Data on mortality of immatures was percent transformed and was subjected to probit analysis (Finney et al., 1971) to calculate LC_50_ and LT_50_ values. SAS 9.2 software was used for all data analysis (SAS Institute, 1988).

## Results

### Characterization of *C. fumosorosea*-BC Nanoparticles

**FIGURE 1:**
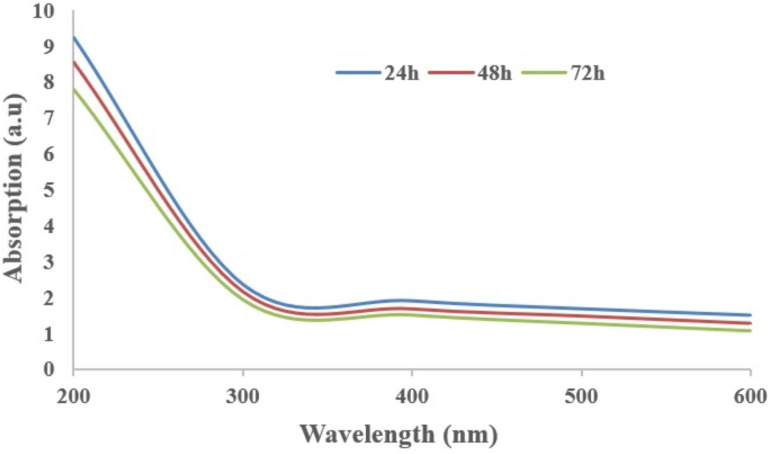
Absorption spectra of *Cordyceps fumosorosea*-biochar nanoparticles of different wavelengths at different time intervals.

**FIGURE 2:**
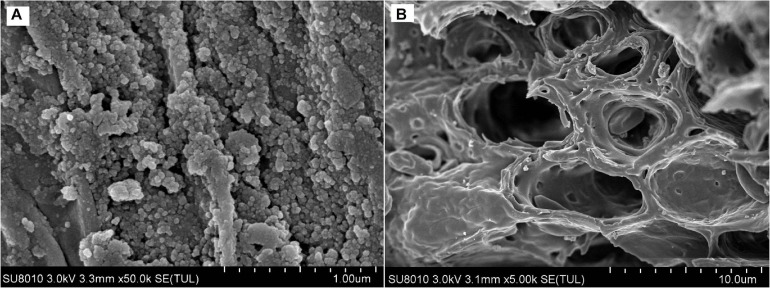
Scanning Electron Microscopy (SEM) of biochar particles and *Cordyceps fumosorosea*-Biochar nanoparticles. **(A)** 1.00 micrometer; **(B)** 10.0 micrometer.

**FIGURE 3:**
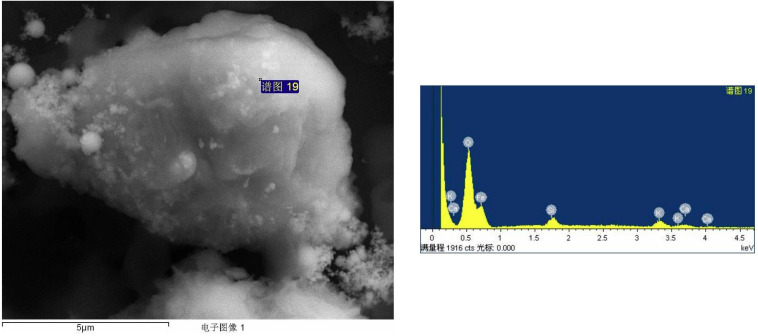
High Resolution Transmission Electron Microscopy (HRTEM) and Energy dispersive X-ray spectroscopy profile of biochar particles and *Cordyceps fumosorosea*-Biochar nanoparticles.

**FIGURE 4:**
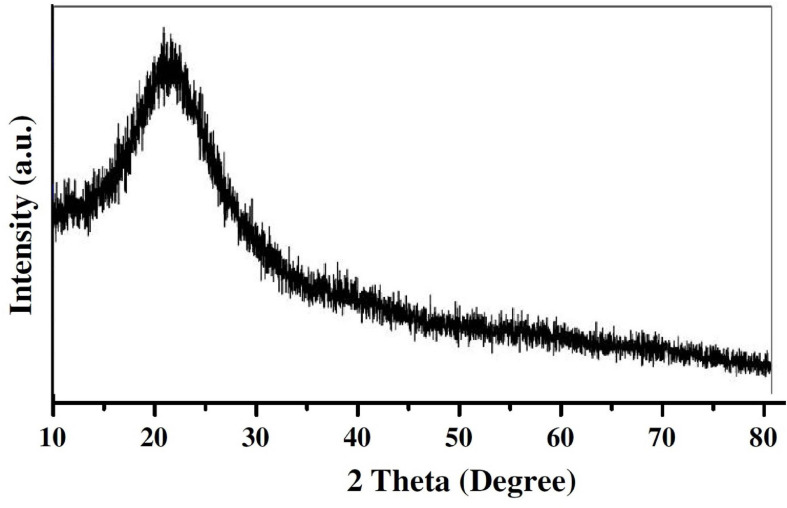
X-ray diffraction pattern of *Cordyceps fumosorosea*-biochar nanoparticles.

The supplementation of biochar into conidial filtrate of *C. fumosorosea* induced a reduction process resulting in a change of the suspension’s color (black to dark color) 3 days post incubation which confirmed the formation of *C. fumosorosea*-BC nanoparticles. The UV analysis of aqueous suspension further confirmed the synthesis of *C. fumosorosea*-BC nanoparticles. UV-absorption spectra of samples obtained at different time intervals had a constant reduction in UV spectra at different wavelengths with a specific surface plasmon absorption band between 350 and 400 nm (Figure 1). The SEM images revealed adherence of biochar particles onto the *C. fumosorosea* conidial surface confirming the proper formation of *C. fumosorosea*-BC nanoparticles (Figure 2). HRTEM micrograph depicted well dispersed and spherical shaped biochar particles. The HRTEM of *C. fumosorosea*-biochar revealed granule like structure due to the adherence of biochar particles onto the *C. fumosorosea* conidial surface (Figure 3). X-ray diffractometry analysis of *C. fumosorosea*-BC nanoparticles (dried powder) showed diffracted intensities at 2θ angles ranging from 10° to 80°. The target of XRD analysis was CuKα with a wavelength of 1.5406 Å. The X-ray diffractogram showed multiple strong peaks at 2θ = 20°–25° which were possibly related to the graphite structure of biochar (Figure 4).

**FIGURE 5:**
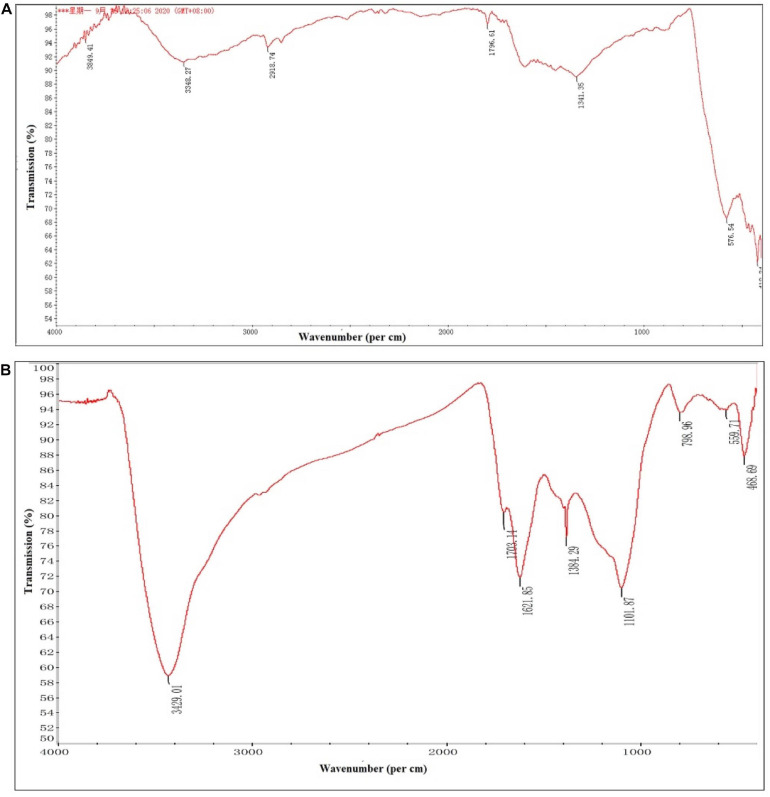
Fourier-transform infrared spectroscopy pattern of biochar particles and *Cordyceps fumosorosea*-biochar nanoparticles. **(A)** Biochar particles. **(B)** *Cordyceps fumosorosea*-biochar nanoparticles.

The FTIR spectroscopic analysis was carried out for identification of biomolecules responsible for the reduction as well as capping of bio-reduced *C. fumosorosea*-BC nanoparticles. The FTIR spectra of *C. fumosorosea*-BC nanoparticles had absorption peaks at 3429.01, 1703.14, 1621.85, 1384.29, 1101.87, 798.96, 559.71, and 468.96 in the region of 4,000–400 cm^–1^ (Figure 5A). The analysis of spectral peaks showed the existence of O–H giving a very strong and sharp beak, C=O showed one small and one medium strong beak, C–O showed a sharp beak, and aromatic ring structure having a very strong structure between amino acid residues and synthesized proteins.

**FIGURE 6:**
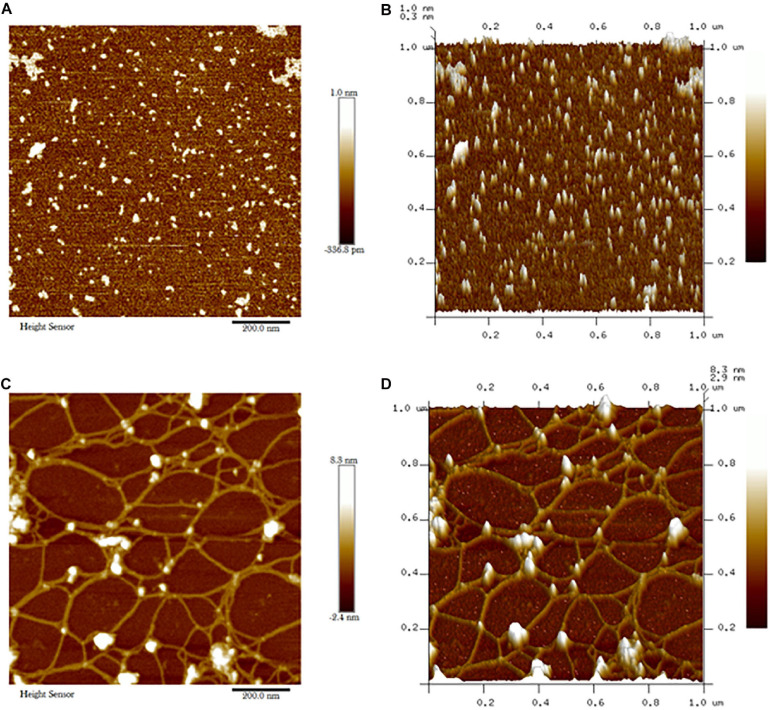
Atomic force microscopy (AFM) of biochar particles and *Cordyceps fumosorosea*-Biochar nanoparticles. **(A)** 2D image of biochar particles. **(B)** 2D image of biochar particles. **(C)** 2D image of *Cordyceps fumosorosea*-biochar nanoparticles. **(D)** 3D image of Cordyceps fumosorosea-biochar nanoparticles.

Atomic force micrograph clearly showed surface topology of biochar and *C. fumosorosea*-biochar nanoparticles. The average size of biochar particle was 24.303 nm whereas the average size of *C. fumosorosea*-biochar nanoparticles was 49.151 nm (Figure 6).

**FIGURE 7:**
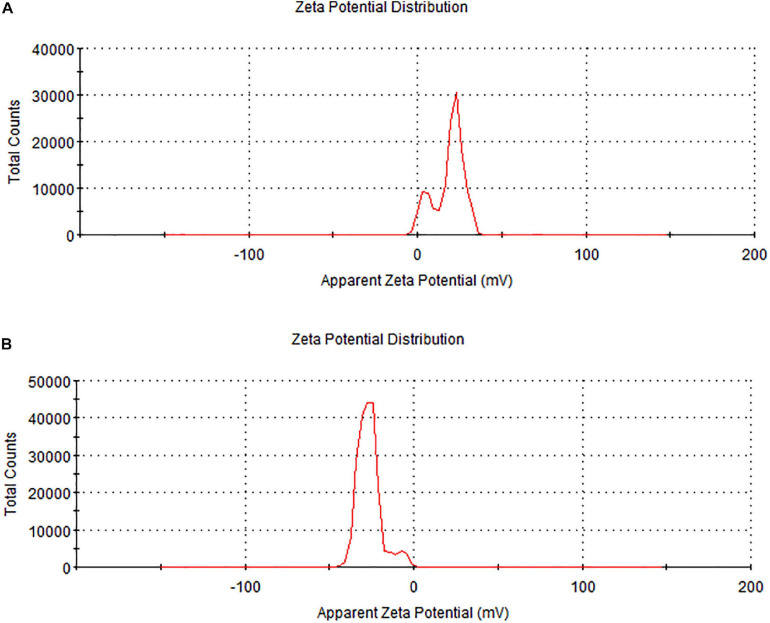
Zeta potential of biochar particles and *Cordyceps fumosorosea*-biochar nanoparticles. **(A)** Biochar particles. **(B)** *Cordyceps fumosorosea*-biochar nanoparticles.

Zeta potential is an important parameter to test the stability of nanoparticles. The zeta potential value of biochar was 18.9 mV while the zeta potential value of *C. fumosorosea* conidia was −24.2 mV. The zeta potential value of *C. fumosorosea*-biochar nanoparticles was −26.3 mV (Figure 7).

### Toxicity of *C. fumosorosea*-BC Nanoparticles Against *Bemisia tabaci*

#### Effects of *C. fumosorosea*-BC Nanoparticles on Egg Hatchability and Larval Development

**FIGURE 8:**
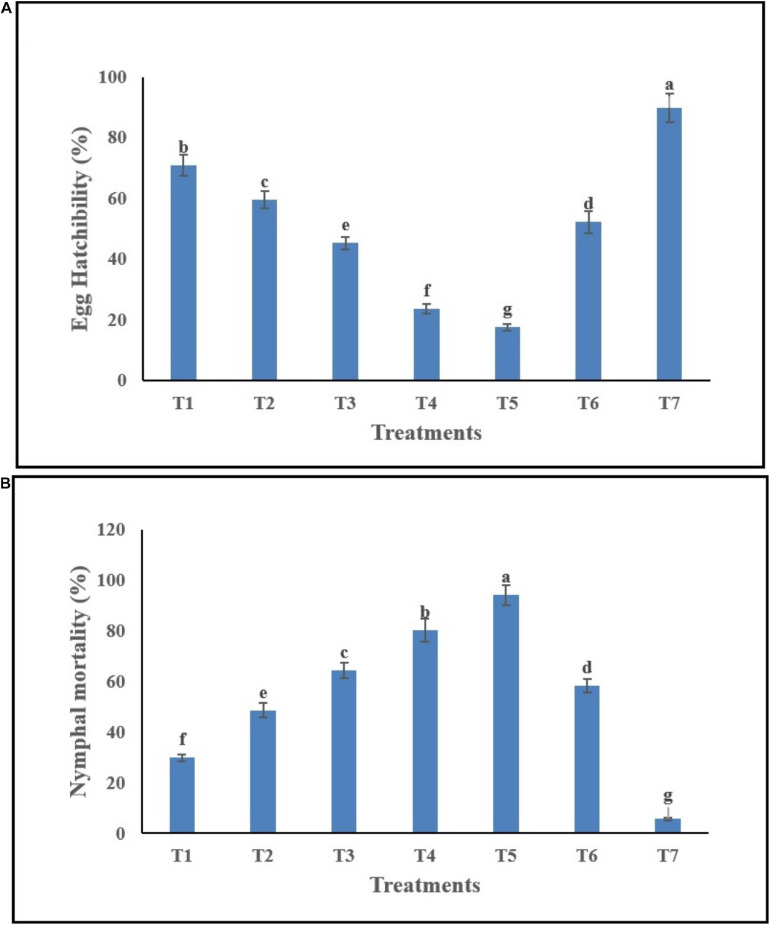
Effects of *C*. *fumosorosea*-biochar nanoparticles on **(A)** egg hatchability and **(B)** nymphal mortality of B. tabaci.*T1 = 5.0 ppm *C*. *fumosorosea*-biochar nanoparticles; T2 = 7.5 ppm *C*. *fumosorosea*-biochar nanoparticles; T3 = 10.0 ppm *C*. *fumosorosea*-biochar nanoparticles; T4 = 20.0 ppm *C*. *fumosorosea*-biochar nanoparticles; T5 = 50.0 ppm *C*. *fumosorosea*-biochar nanoparticles; T6 = 100.0 ppm *C*. *fumosorosea* conidial suspension; and T7 = control.

Egg hatchability (%) of *B. tabaci* in response to *C. fumosorosea*-BC nanoparticle application showed significant differences among different treatments and control 7 days post application (*F*_6_,_14_ = 36.64; *P* < 0.001). The highest egg hatchability (%) of *B. tabaci* was shown under T7 (control) whereas the lowest egg hatchability (%) was observed for T5 (*C. fumosorosea*-BC nanoparticles 50 ppm) (Figure 8A).

Different treatments of *C. fumosorosea*-BC nanoparticles significantly affected the nymphal mortality (%) of *B. tabaci* 7 days post application (*F*_6_,_14_ = 32.81; *P* < 0.001). The highest larval mortality (%) of *B. tabaci* was observed for T5 (*C. fumosorosea*-BC nanoparticles 50 ppm) whereas the lowest larval mortality (%) was observed for T7 (control) (Figure 8B).

#### Microscopic Examination of Egg Infection by *C*. *fumosorosea*-BC Nanoparticles

**FIGURE 9:**
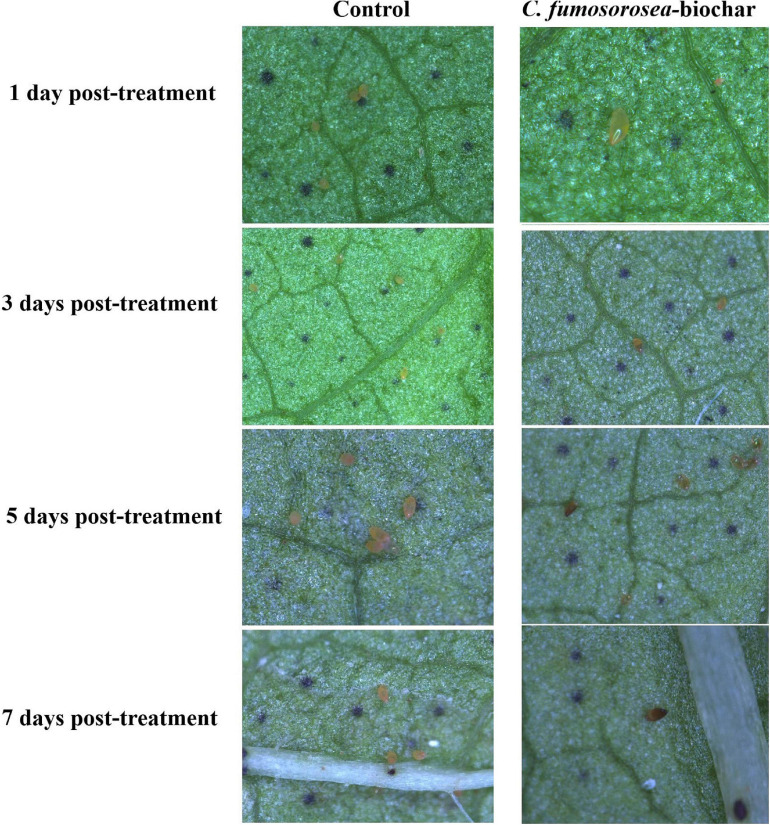
Microscopic explanation of *Bemisia tabaci* egg infection by *Cordyceps fumosorosea*-biochar nanoparticles.

*Bemisia tabaci* eggs infected by *C*. *fumosorosea*-BC nanoparticles had little change in color but appeared shrunk compared with controls 3 days post treatment when observed under the microscope. After 7 days of treatment, the eggs became clearly shrunk and dark colored. The symptoms of egg infection by all concentrations of *C*. *fumosorosea*-BC nanoparticles started to appear after 3 days of infection and increased until the 7th day (Figure 9).

#### Toxicity of *C. fumosorosea*-BC Nanoparticles Against Immature Instars of *Bemisia tabaci*

**FIGURE 10:**
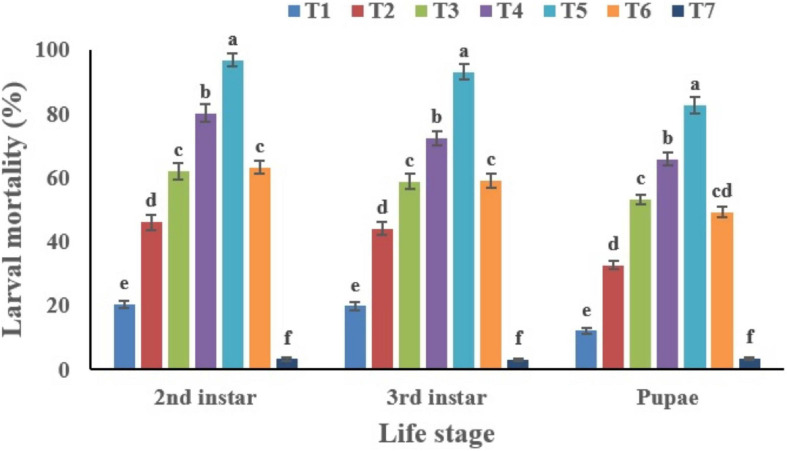
Percentage mortality of *Bemisia tabaci* immatures in response to different treatments of *Cordyceps fumosorosea*-biochar nanoparticles and control after 7 days. *T1 = 5.0 ppm *C*. *fumosorosea*-biochar nanoparticles; T2 = 7.5 ppm *C*. *fumosorosea*-biochar nanoparticles; T3 = 10.0 ppm *C*. *fumosorosea*-biochar nanoparticles; T4 = 20.0 ppm *C*. *fumosorosea*-biochar nanoparticles; T5 = 50.0 ppm *C*. *fumosorosea*-biochar nanoparticles; T6 = 100.0 ppm *C*. *fumosorosea* conidial suspension; and T7 = control.

**FIGURE 11:**
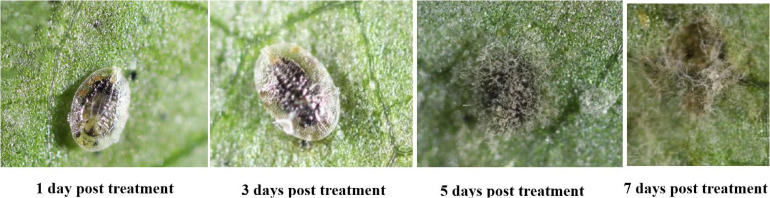
Images of *Bemisia tabaci* infection by *Cordyceps fumosorosea*-biochar nanoparticles at 1, 3, 5, and 7 days post-inoculation.

The mortality of *B. tabaci* immatures (2nd instar nymphs, 3rd instar nymphs and pupae) increased in a dose dependent manner (Figure 10). The highest mortality was recorded for the second instar nymphs (96.84%). The cumulative mortality values of the second instar, third instar and pupal stages were 96.84, 93.27, and 82.74%, respectively for the highest concentration of *C. fumosorosea*-BC nanoparticles (50 ppm). The cumulative mortality for the lowest nanoparticle concentration (5.0 ppm) recorded after 7 days was 20.49, 19.87, and 12.16% for the second instar, third instar and pupal stages, respectively. Second instar nymphs of *B. tabaci* were most susceptible among the life stages observed during this study. Immatures of whitefly (2nd instar) treated with *C. fumosorosea*-BC nanoparticles became slightly dark colored after 1 day of treatment and growth of fungus was evident on insect cadavers after 5 days of treatment (Figure 11).

Our findings revealed that *C. fumosorosea*-BC nanoparticles showed considerable pathogenicity against immature life stages of *B. tabaci* with higher LC_50_ values against pupa compared to the second and third nymphal instars (Table 1). The LC_50_ values of *C. fumosorosea*-BC nanoparticles against second instar nymphs, third instar nymphs and pupa were 6.80, 7.45, and 8.64 ppm, respectively.

**TABLE 1:** Median lethal concentration (LC_50_) values of *Cordyceps fumosorosea*-BC nanoparticles against immature life stages of *Bemisia tabaci*.

Life stage	Mortality function	LC_50_ (ppm)	*R*^2^
2nd instar nymph	1.63 + 4.03x	6.80 ± 0.89 b	0.994
3rd instar nymph	2.24 + 3.16x	7.45 ± 0.96 ab	0.998
Pupa	2.58 + 2.57x	8.64 ± 0.93 a	0.969

*Means in the same column followed by different letters are significantly different from each other (Tukey’s *P* < 0.05).*

The highest median lethal time (LT_50_) values of *C. fumosorosea*-BC nanoparticles (4.07 days) were observed for pupae whereas the lowest LT_50_ (3.25 days) was recorded for second nymphal instars when treated with *C. fumosorosea*-BC nanoparticles at a concentration of 20 ppm (Table 2).

**TABLE 2:** Median lethal time (LT_50_) values of *Cordyceps fumosorosea*-BC nanoparticles against immature life stages of *Bemisia tabaci.*

Life stage	Mortality function	LT_50_ (days)	*R*^2^
2nd instar nymph	3.85 + 2.23x	3.25 ± 0.29 c	0.984
3rd instar nymph	3.45 + 2.69x	3.69 ± 0.52 b	0.988
Pupa	3.70 + 2.12x	4.07 ± 0.51 a	0.994

*Means in the same column followed by different letters are significantly different from each other (Tukey’s *P* < 0.05). Each batch (*N* = 30) of immatures was treated with a 50 ppm concentration of nanoparticles.*

## Discussion

The application of nanotechnology in combination with insect pathogens can be a promising biological alternative to synthetic chemicals for insect pest management (Sabbour and El-Aziz, 2015). Nanoparticles/nanomaterials-based formulations of insect pathogens have been recently tested insecticides for effective management of different insect pests (Rai and Ingle, 2012). Entomopathogenic fungi are mostly mixed with different metals (Ag, Cu, Au, or Fe) for the preparation of nano-formulations (Gajbhiye et al., 2009; Goswami et al., 2010). During our previous studies, *I. fumosorosea*-iron nanoparticles (100 ppm) showed 68% reduction in egg hatchability as well as 98.30, 92.16, and 80.33% mortality of first, second, and third instar *B. tabaci* nymphs respectively (Wang et al., 2019).

The present work reports the synthesis of *C. fumosorosea-*BC nanoparticles and their application for *B. tabaci* management. The treatment of *C. fumosorosea* with biochar under dark conditions resulted in change of filtrate color from light to dark black showing the synthesis of *C. fumosorosea-*BC nanoparticles. The visualization of a characteristic peak between 350 and 400 nm in UV profile further confirmed the *C. fumosorosea-*BC nanoparticles. Our findings are different from Wang et al. (2019) who reported a similar change in color of culture filtrate and a characteristic peak at 470 nm during biosynthesis of *I. fumosorosea*-Fe^0^NPs. These findings are also in line with the results of Banu and Balasubramanian (2014) who also observed similar changes in culture filtrates during the synthesis of silver-based nanoparticles of *I. fumosorosea*. The color change during extracellular synthesis of *C. fumosorosea-*BC nanoparticles can be related/explained by two phenomena: (i) nitrate utilization through reduction of nitrate and ammonia during bio-reduction of biochar (Hussain et al., 2016); and (ii) the excitation of surface plasmon vibration of nanoparticles (Vigneshwaran et al., 2007). The existence of biochar on *C. fumosorosea* conidial surface was further confirmed through scanning electron micrographs. The XRD analysis showed characteristic peaks between 2θ values of 20°–25° corresponding to a spherical structure calculated through Bragg’s law (Bragg and Bragg, 1913) and Miller indices (Miller, 2009). The characteristic peaks between 2θ values of 20°–25° are similar to the XRD pattern of pure biochar which shows characteristic peaks between 2θ values 20°–25° (Hussain et al., 2016). The Fourier transformation infra-red spectrum also confirmed the presence of different biomolecules, for example, proteins which can be responsible for the synthesis of *C. fumosorosea-*BC nanoparticles (Yan et al., 2015). AFM was used as the principal method to screen the dissolution and agglomeration pattern of nanoparticles. The topographical image of biochar and *C. fumosorosea*-biochar nanoparticles in particular bright spots indicates the formed nanoparticles. The average size of *C. fumosorosea*-biochar nanoparticles was 49.151 nm which is lower than the average size of *Nomuraea rileyi* metabolite-chitosan nanoparticles observed by Namasivayam et al. (2018). This variation in nanoparticle size can be related to the size of materials used for nanoparticles since the size of biochar particles (24.03 nm) is lower than the size of chitosan (50.06 nm) observed by Namasivayam et al. (2018). Zeta potential is the important parameter which influences their stability in suspension. The communal repulsion of nanoparticles hinges on having either a large negative or positive zeta potential (Zain et al., 2014). Zeta potential values *C. fumosorosea*-biochar nanoparticles were in the negative range.

A potential microbial pest control agent should have high infection rate and should readily kill all life stages (feeding and non-feeding or mobile and immobile) of the target insect pest. During this study, different concentrations of *C. fumosorosea-*BC nanoparticles caused significant reduction in hatchability of *B. tabaci* eggs. Our results further showed that even the lowest concentration of *C. fumosorosea-*BC nanoparticles caused reduction in egg hatchability when compared with the control. Furthermore, the whitefly nymphs emerging from the eggs treated with different concentrations of *C. fumosorosea-*BC nanoparticles were also affected by *C. fumosorosea-*BC nanoparticles. The possible mechanism of reduction in egg hatchability can be related to the mode of infection of *C. fumosorosea* in which its target host is penetrated, followed by desiccation and eventual death of the host (Huang et al., 2010).

The current findings indicate that susceptibility of whitefly life stages to *C. fumosorosea-*BC nanoparticles was as follows; 2nd instar nymph > 3rd instar nymphs > pupa, which is consistent with findings of previous studies on the pathogenicity of metal-based *I. fumosorosea* nanoparticles (Banu and Balasubramanian, 2014; Wang et al., 2019). The indices of probit analysis such as median LC_50_ and LT_50_ are common parameters to gauge the effectiveness of a pesticide. The dose-mortality relationship of *B. tabaci* immature life stages (2nd instar nymphs, 3rd instar nymphs and pupa) to nanoparticles showed increased mortality in a dose dependent manner. The LC_50_ values of *C. fumosorosea-*BC nanoparticles against 2nd instar nymphs, 3rd instar nymphs and pupa were 6.80, 7.45, and 8.64 ppm, respectively. Wang et al. (2019) reported LC_50_ values of 19.17, 26.10, and 37.17 ppm for *I. fumosorosea* Fe^0^NPs against 2nd instar nymphs, 3rd instar nymphs and pupa of *B. tabaci*, respectively. Banu and Balasubramanian (2014) found the LC_50_ value of 0.79 ppm for *B. bassiana* AgNPs against 2nd nymphal instar of *Aedes aegypti*. The median lethal time (LT_50_) values of *C. fumosorosea-*BC nanoparticles applied at the rate of 20 ppm against 2nd instar nymphs, 3rd instar nymphs and pupa of *B. tabaci* were 3.25, 3.69, and 4.07 days, respectively. The LT_50_ values of this study are a little higher than those observed by Wang et al. (2019) who reported a LT_50_ value of 3.15, 3.37, and 4.22 days for 100 ppm concentration of *C. fumosorosea* Fe^0^NPs against 2nd instar nymphs, 3rd instar nymphs and pupa of *B. tabaci* following 7 days of fungal treatment. The possible mechanism of action of *C. fumosorosea*-BC nanoparticles can be explained by different phenomenons or hypothesis such as: (1) enhanced degradation of the insect cuticle or delayed ecdysis in response to *C. fumosorosea*-BC due to a combined action of extracellular hydrolytic enzymes produced by fungi known for their effect on an insect cuticle (Ali et al., 2009, 2010a) and biochar particles known to degrade hydrocarbons in nature (Ahmad et al., 2014) so they therefore possibly degrade alkanes present in an insect cuticle; (2) possible degradation of the insect gut peritrophic membranes by chitinase produced by the fungi (Brandt et al., 1978; Shahabuddin et al., 1993; Ali et al., 2010b) and also the degradation of the peritrophic membrane being further enhanced by the addition of biochar which is known for its ability to degrade hydrocarbons and aromatic structures in nature (Ahmad et al., 2014); and (3) decreased efficiency to convert the consumed food into a growth and energy source through possible diversion of energy from growth to the detoxification process (Ali et al., 2010b; Xu et al., 2020).

## Concluding Remarks

In summary, the above results suggest that *C. fumosorosea-*BC nanoparticles are toxic against *B. tabaci* immatures and that *C. fumosorosea-*BC nanoparticles can potentially be used within biorational *B. tabaci* management programs. However, further work is required to determine the efficacy and durability of *C. fumosorosea-*BC nanoparticles under field conditions.

## Data Availability Statement

The raw data supporting the conclusions of this article will be made available by the authors, without undue reservation.

## Author Contributions

XW and SA conceived and designed the research. JX and TS conducted the experiment. SA wrote the manuscript. All authors read and approved the manuscript.

## Conflict of Interest

The authors declare that the research was conducted in the absence of any commercial or financial relationships that could be construed as a potential conflict of interest.
